# Imbalance of the redox system and quality of tilapia fillets subjected to pre-slaughter stress

**DOI:** 10.1371/journal.pone.0210742

**Published:** 2019-01-15

**Authors:** Elenice Souza dos Reis Goes, Marcio Douglas Goes, Pedro Luiz de Castro, Jorge Antônio Ferreira de Lara, Ana Carolina Pelaes Vital, Ricardo Pereira Ribeiro

**Affiliations:** 1 School of Agrarian Sciences, Federal University of Grande Dourados, Dourados, Mato Grosso do Sul, Brazil; 2 Animal Science Post-Graduate Program, State University of Western Paraná, Marechal Cândido Rondon, Paraná, Brazil; 3 Animal Science Post-Graduate Program, State University of Maringá, Maringá, Paraná, Brazil; 4 Brazilian Agricultural Research Corporation, EMBRAPA Pantanal, Corumbá, Mato Grosso do Sul, Brazil; 5 Food Science Post-Graduate Program, State University of Maringá, Maringá, Paraná, Brazil; 6 Department of Animal Science, State University of Maringá, Maringá, Paraná, Brazil; University of Illinois, UNITED STATES

## Abstract

The objective of this study was to evaluate the effect of oxidative stress on the instrumental and sensory quality of Nile tilapia fillets. The experiment was conducted in a 2x2 factorial arrangement, evaluating densities (60 and 300 kg m^-3^) and depuration times (1 and 24 hours) in a total of four treatments. The serum levels of cortisol and gene expression levels of catalase (CAT), glutathione peroxidase (GPx) and 70 kDa heat shock protein (HSP70) as well as the pH, color, tenderness, water-holding capacity and sensory analysis of the fillets were evaluated. High density (300 kg m^-3^) resulted in higher mean cortisol levels, lower expression of CAT and GPx enzymes as well as higher expression of HSP70. Fish under this treatment also exhibited fillets with greater tenderness, higher lightness, lower redness and lower sensory acceptance. The longer depuration time (24 hours) resulted in lower expression of the CAT and GPx enzymes and fillets with higher lightness. The water-holding capacity was not affected by the different treatments. Therefore, low density and longer depuration times are recommended for decreased stress and improved quality of fillets.

## Introduction

Stress is a condition of high aerobic energy demand to supply the body's maintenance mechanisms during activation for adaptation and resistance of the body to stressful conditions [1]. In aquaculture, fish are subjected both to acute stressors, such as handling, and chronic stressors, including environmental changes (such as temperature, water quality and salinity), interactions with other fish and prolonged physical stress (such as transport and increased densities) [2].

When fish are subjected to stress, vigorous swimming increases anaerobic glycolysis, leading to lactic acid production and a consequent decline in muscle pH, which is accompanied by a faster onset of rigor mortis [3] [4] [5]. The combination of stress and intense physical activity at pre-slaughter can increase the degree of protein denaturation and, thus, increase the access of proteolytic enzymes to protein substrates, leading to faster muscle softening, which is detrimental for fish muscle [6].

In addition to denaturation and proteolysis, muscle proteins also undergo oxidative damage after slaughter and subsequent meat aging [7]. Protein oxidation is responsible for many biological changes, such as protein fragmentation or aggregation and decreased protein solubility, which affects meat quality [8]. Oxidation may also play a role in controlling the proteolytic activity of enzymes and may be linked to meat tenderness [9]. However, endogenous antioxidant factors, such as enzymes, control oxidation in muscle tissues [8]. Enzymes, such as superoxide dismutase (SOD), catalase (CAT) and glutathione peroxidase (GPx), can neutralize meat oxidation [10]. A recent study has shown that CAT, GPx and SOD activities are significantly lower in pale, soft and exudative (PSE) chicken meat, which makes this type of meat more susceptible to proteolysis and protein oxidation [11].

Thus, oxidative stress may be an important cell mechanism in the process of meat softening [12]. Oxidative metabolism has also been cited as a potential controlling factor of heat shock proteins (HSPs), especially because it protects structural proteins against oxidative stress and proteolysis, which are essential for tenderness [13] [12]. HSPs can act as molecular chaperones, facilitating protein folding, preventing protein aggregation, or conducting improperly folded proteins into specific degradation pathways [14], and HSPs also play a role in the refolding of damaged proteins for protection and repair of cells and tissues [15]. A variety of stresses, including oxidative stress, have been linked to increased HSP expression in skeletal muscle [14] [15].

The relationship between redox imbalance and meat quality in fish subjected to pre-slaughter stress is still unknown. Therefore, this study aimed to evaluate the effect of depuration-related oxidative stress on the instrumental and sensory quality of Nile tilapia fillets.

## Materials and methods

Method was carried out in accordance with the guidelines of the Brazilian College for Animal Experimentation (COBEA; http://www.cobea.org.br) and was approved by the Committee on Animal Care of the Universidade Estadual de Maringá—Brazil.

### Animals and experimental design

Experimental animals were obtained from farming in cages in the Corvo River, Diamante do Norte Municipality, Paraná (PR), Brazil (22°39' S, 052°46' W). Nile Tilapia of the Tilamax variety (±800 g) were transported from the Corvo River to the UEM/CODAPAR Aquaculture Station in the municipality of Maringá-PR where they were placed in 10 m^3^ concrete tanks at a density of 5 kg m^-3^. The fish were kept in these tanks for 40 days to recover from the stress related to transportation and for adaptation of the animals to the experimental structure.

After this period, an experiment was conducted in a 2x2 factorial arrangement using density (60 and 300 kg m^-3^) and depuration time (1 and 24 hours) as experimental factors with a total of 4 treatments with 20 replicates per treatment (where the fish was the experimental unit).

Initially, the animals were removed from the concrete tanks with the aid of a hand net, and placed in 500 L polyethylene tanks equipped with water recirculation and an artificial aeration system, and one box was used per treatment. The fish were subjected to the following treatments: 60 kg m^-3^ for 1 hour; 60 kg m^-3^ for 24 hours; 300 kg m^-3^ for 1 hour; and 300 kg m^-3^ for 24 hours. We sampled 20 fish per treatment. Of these, 5 fish were used for blood, muscle and liver collection for cortisol analysis and gene expression, after which, these animals were euthanized and filleted for meat quality analyzes. Instrumental quality analyzes of fillets were performed on 10 fish per treatment (including animals submitted to blood and tissue samples). Sensory analysis was performed with the remaining 10 fish per treatment.

Animals in each treatment were euthanized by severing the spinal cord followed by hand filleting. Whole skinless fillets were washed in chlorinated water at 5 ppm, vacuum packed and transported on ice to the laboratory for meat quality analyses.

### Cortisol levels

From each fish (n = 5), 2 mL of blood was collected by caudal puncture using disposable syringes. The serum was obtained after submerging the blood samples in a water bath at 37°C for 10 minutes followed by centrifugation at 1000 x g for 10 minutes and collection of the supernatant (serum). Serum cortisol concentration was measured using a chemiluminescent magnetic microparticle immunoassay (CMIA) on the Architect Ci8200 platform using a kit (Abbott Laboratories, Abbott Park, Ill., USA).

### Gene expression

To evaluate gene expression levels of glutathione peroxidase (GPX), catalase (CAT) and 70 kDa heat shock protein (HSP70), samples of approximately 2 g of white muscle and liver were collected from five fish per treatment. The samples were stored in liquid nitrogen until analysis.

Total RNA was extracted using Trizol (Invitrogen, Carlsbad CA, USA) according to the manufacturer's instructions at a ratio of 1 mL for each 70 mg of tissue. All materials used were pretreated with RNase inhibitor—RNase AWAY (Invitrogen, Carlsbad, CA, USA).

To evaluate total RNA concentration, samples were measured by the fluorometric method using the Qubit RNA BR Assay Kit (Invitrogen, Carlsbad CA, USA). RNA integrity was evaluated using a 1% agarose gel stained with SYBR Safe DNA Gel Stain (Invitrogen, Carlsbad CA, USA) and visualized on a UV transilluminator.

To remove possible genomic DNA residues, RNA samples were treated with QuantiNova gDNA Removal Mix (Qiagen GmbH, Hilden, Germany) at 45°C for 2 minutes according to the manufacturer's instructions. After removal of genomic DNA, 5 μg of RNA was used for cDNA synthesis using the QuantiNova Reverse Transcription Kit (Qiagen GmbH) according to the manufacturer's protocol. In a sterile tube, 5 μg of total RNA, 1 μL of QuantiNova Reverse Transcription Enzyme and 4 μL of QuantiNova Reverse Transcription Mix were added. The reverse transcriptase reaction was incubated for 3 minutes at 25°C followed by 45°C for 10 minutes and subsequent inactivation for 5 minutes at 85°C. The reaction was then immediately placed on ice. Samples were stored at -20°C until analysis.

qPCR analyses were performed using a Step One Plus Real-Time PCR System (Applied Biosystems, Carlsbad, CA, USA) using the QuantiNova SYBR Green PCR Kit in duplicate. A total reaction volume of 20 μL was used, containing 10 μL of 2X SYBR Green PCR Master Mix, 2 μL of QN Rox Reference Dye, 0.8 μL of each primer (400 nM), 5 μL of cDNA (400 ng) and 1.4 μL of RNAse-free water.

The reactions were subjected to the following thermocyler protocol: 95°C for 2 minutes; 40 cycles of 95°C for 5 seconds and annealing/extension at 60°C for 10 seconds. The melting curve was obtained to determine the specificity of the reactions.

For real-time PCR (qRT-PCR) analyses of GPX, CAT and HSP70 genes, primers were designed according to the sequences deposited at www.ncbi.nlm.nih.gov for *Oreochromis niloticus* using the www.idtdna.com website (Table 1). The β-actin gene was used as an endogenous control, and the primer developed by Yang et al. [16] was selected.

**Table 1:** Characteristics of the primers used in this study.

Primer	Sequence	Amplicon size (bp)	Accession number
GPX	F: CGCCGAAGGTCTCGTTATTT R: TCCCTGGACGGACATACTT	107	NM_001279711.1
CAT	F: CCCAGCTCTTCATCCAGAAAC R: GCCTCCGCATTGTACTTCTT	103	JF801726.1
HSP70	F: TCACCATCACCAACGATAAGG R: TCCTCGGCTTTGTATTTCTCTG	85	FJ213839.1
β-actin	F: TGGTGGGTATGGGTCAGAAAG R: CTGTTGGCTTTGGGGTTCA	217	XM_003455949

The qRT-PCR results were transformed as suggested by Coble et al. [17] by calculating the adjusted cycle threshold (Ct) using the following equation:

Adjusted Ct = 40 - [(mean Ct of the target gene) + (median Ct of the endogenous gene—mean Ct of the endogenous gene) × (slope of the target gene/slope of the endogenous gene)].

The primers for the genes analyzed proved to be suitable for real-time PCR analysis. The amplification efficiency was similar for the genes of interest, ranging from 90 to 110%. The analysis of the melting curves did not reveal the presence of non-specific products or the formation of primer dimers, which indicated the reliability of the mRNA expression data for the genes studied.

In order to determine the effectiveness of the use of β-actin as an endogenous control, the results obtained for β-actin Ct were submitted to analysis of variance using the Factorial ANOVA procedure in the STATISTICA 7.1 software (Statsoft Inc., Tulsa, OK, USA), at a level of 5% significance. Statistical analysis of β-actin as an endogenous control did not show a significant difference (P>0.05) between the treatments, thus proving its effective use as an endogenous control.

### pH, color, tenderness and water-holding capacity analyses

The pH, color and tenderness (measuring the cutting resistance—shear force) were evaluated 2 hours after slaughter in ten fillets per treatment. For the water-holding capacity and sensory analyses, the fillets were stored under freezing (-18±2°C) until analysis.

The pH was measured at three different points (dorsal, central and ventral parts) of each fillet, in all fillets, using a portable digital potentiometer (Mettler Toledo model 1140) with puncture electrode for measurement in meats.

The color was evaluated on the ventral side of the fillet, and six different readings per sample were recorded. The following values of lightness (L*) were evaluated using a colorimeter (Minolta model CR-10) at a 90° angle at room temperature according to the CIELAB system: L* defines the lightness (L* = 0 black and L* = 100 white), chroma a* (red-green component), and chroma b* (blue-yellow component).

The fillet tenderness was analyzed by measuring the cutting resistance (shear force) using a Brookfield texture analyzer-CT III, which was equipped with a SMS shear cell (Stable Micro Systems) and a Warner Bratzler Blade (USDA; thickness of 3 mm, length of 70 mm and angle of 60°). Prior to analysis, the fillets were kept at room temperature for approximately 1 hour. The fillets were cut into cubes measuring approximately 20 x 25 x 20 mm, cutting transversely to the direction of the muscle fibers. The analysis was performed in triplicate per fillet, and the shear force was expressed in gram-force (gf). The maximum peak force (gf) required to shear through the sample was recorded as shear force, and was regarded as the tenderness of the fillet.

The water-holding capacity (WHC) was evaluated according to Lankhmanan et al. [18]. For this purpose, thawed fillet samples (n = 10) were weighed on an analytical balance. Triplicate samples of 1 g of raw fillet were placed in 1.5 mL tubes lined with filter paper. The tubes were centrifuged at 1318 x g for 4 minutes at 4°C. Samples were weighed after centrifugation and oven dried at 70°C for 12 hours. The dried samples were again weighed. The following formula was used to calculate the WHC:

$$WHC\%=\frac{PCSW-DSW}{ISW}\times 100$$ (1)

where WHC% is the water-holding capacity; ISW is the initial sample weight; PCSW is the post-centrifugation sample weight; and DSW is the dry sample weight.

### Sensory analysis

For sensory analysis, tilapia fillets were thawed at 4°C for 24 hours. Each fillet was then individually wrapped with aluminum foil and cooked on a preheated grill (Philco Jumbo InoxGrill, Philco SA, Brazil) at 200°C until reaching an internal temperature of 90°C as monitored with a food thermometer (Incoterm, 145 mm, Incoterm LTDA, Brazil). Each fillet was cut into eight 2 x 2 cm cubes and kept warm (50°C) until consumer evaluation (less than 10 minutes after cooking).

Consumption tests were performed in a private room properly adapted to perform a sensory test. One hundred and twenty consumers were randomly selected among students, staff and visitors.

Twelve sessions were held, and each session had ten different consumers. Each consumer evaluated four samples coded with a random code of three digits per session, corresponding to the different treatments. Samples were provided in a random order to avoid order and transposition effects [19] (Macfie et al., 1989). Consumers were asked to taste and evaluate each sample regarding the acceptability of 4 attributes (color, texture, juiciness and overall acceptability) using a 9-point scale, ranging from 1 (disliked extremely) to 9 (liked extremely). The mean value of the scale was not included as described by Font i Furnols et al. [20]. Consumers were asked to eat crackers and rinse their mouths with water before evaluating each sample, including the first sample.

### Statistical analysis

The results were subjected to analysis of variance using the Factorial ANOVA procedure in the STATISTICA 7.1 software (Statsoft Inc., Tulsa, OK, USA). The effects of density, depuration time and the interaction between the factors were evaluated at a significance level of 5%. In the case of significant differences (P<0.05), Tukey’s test was applied to test for differences between means. All data are expressed as the mean ± standard error of the mean.

For the sensory analysis data, the acceptability of the sensory attributes was verified through analysis of variance using a general linear model (GLM) with the help of SPSS software (v.23.0) for Windows. The treatments (density and depuration time) were considered fixed factors, and consumers were included as random effect. Differences between means were evaluated using Tukey's test (P≤0.05). Principal component analysis (PCA) was used to identify relationships between treatments and sensory attributes and was shown in plot form. The correlation between the attributes was evaluated using Pearson’s correlation. Both analyses were performed using XLSTAT software (v.19.4).

## Results

### Serum cortisol levels and antioxidant gene expression

**Fig 1:**
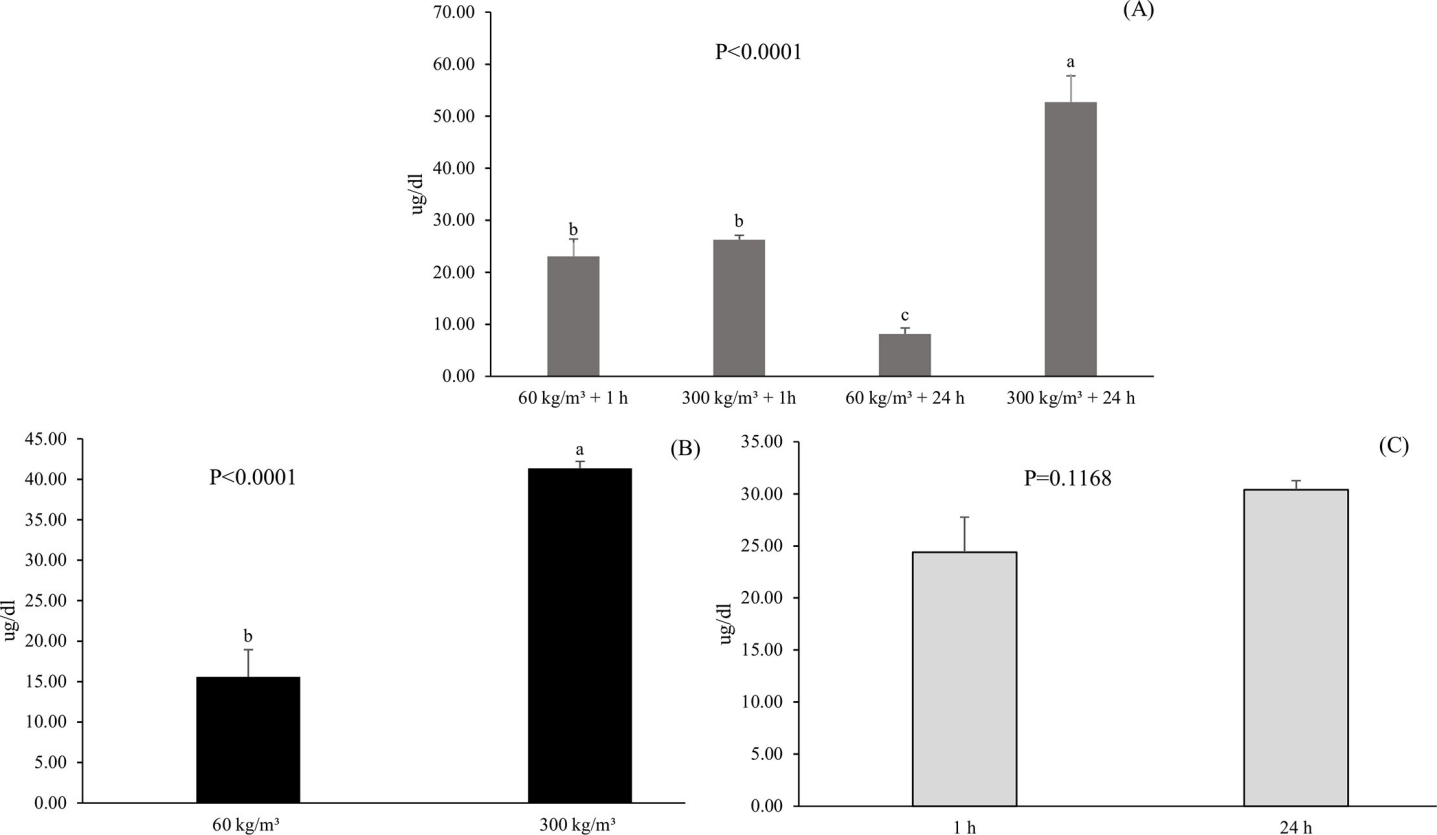
Serum cortisol levels of Nile tilapia at different densities (60 and 300 kg m^-3^) and depuration times (1 and 24 hours). Effects of the interaction density x depuration time (A) and the individual factors (B and C). Lower case letters indicate a significant difference (P<0.05) by Tukey's test.

For serum cortisol levels, there was an effect of the interaction density *versus* depuration time (P = 0.0008) where 300 kg m^-3^ for 24 hours resulted in the highest mean cortisol levels (Fig 1). The lowest level of stress was observed in fish subjected to 60 kg m^-3^ for 24 hours of depuration. The densities of 60 kg m^-3^ and 300 kg m^-3^ for 1 hour of depuration resulted in equal levels of serum cortisol. When the effect of density alone was evaluated, a higher level of cortisol (P<0.0001) was observed in fish subjected to the highest density (300 kg m^-3^).

Regarding the expression of genes involved in the antioxidant defense system, no effect of the interaction (P>0.05) between density and depuration time was observed for the analyzed genes (Table 2) in the liver of tilapias. However, when the factors were individually analyzed, the density of 300 kg m^-3^ resulted in a higher mean CAT expression (P<0.0001). In addition, the longer depuration time (24 hours) resulted in higher mean CAT (P = 0.0003) and GPx mRNA expression (P = 0.0025).

**Table 2:** mRNA expression of antioxidant enzymes in Nile tilapia subjected to different densities (60 and 300 kg m^-3^) and depuration times (1 and 24 hours).

		LIVER		MUSCLE		
Density (kg m^-3^)	Time (h)	Catalase	Glutathione peroxidase	Catalase	Glutathione peroxidase	HSP70
60	1	18.44±0.23	10.42±0.31	13.10±0.24	12.91±0.10a	8.05±0.36
	24	16.22±0.34	8.43±0.53	13.39±0.80	11.03±0.24b	9.75±0.96
300	1	15.18±0.68	10.53±0.71	11.75±0.61	11.50±0.33b	10.88±0.59
	24	12.36±0.13	8.04±0.43	11.77±0.42	11.05±0.29b	10.50±0.37
Density (kg m^-3^)						
60		17.33±0.53 a	9.43±0.52	13.25±0.38 a	11.97±0.44 a	8.90±0.60 b
300		13.77±0.70 b	9.29±0.67	11.76±0.33 b	11.27±0.22 b	10.69±0.32 a
Time (h)						
1		16.81±0.80 a	10.48±0.35 a	12.43±0.42	12.20±0.35 a	9.47±0.70
24		14.29±0.88 b	8.24±0.32 b	12.58±0.54	11.04±0.17 b	10.13±0.49
Effects						
Density vs. time		0,4789	0.6397	0.8157	0.0235	0,1334
Density		0.0000	0.7956	0.0286	0.0262	0.0204
Time		0.0003	0.0025	0.7875	0.0019	0.3173

Data are expressed as the mean ± standard error. Means in the same column followed by different letters differed by Tukey's test (P<0.05).

When the same antioxidant system genes were evaluated in tilapia muscle (Table 2), there was an effect of the interaction between density and depuration period only for GPx mRNA expression (P = 0.0235) where 300 kg m^-3^ for 24 hours of depuration resulted in greater expression compared to the other treatments. When the factors were individually analyzed, the density of 300 kg m^-3^, compared to the density of 60 kg m^-3^, resulted in higher expression of the CAT (P = 0.0286) and GPx (P = 0.0262) enzymes as well as higher expression of HSP70 (P = 0.0204).

### Meat quality parameters

**Fig 2:**
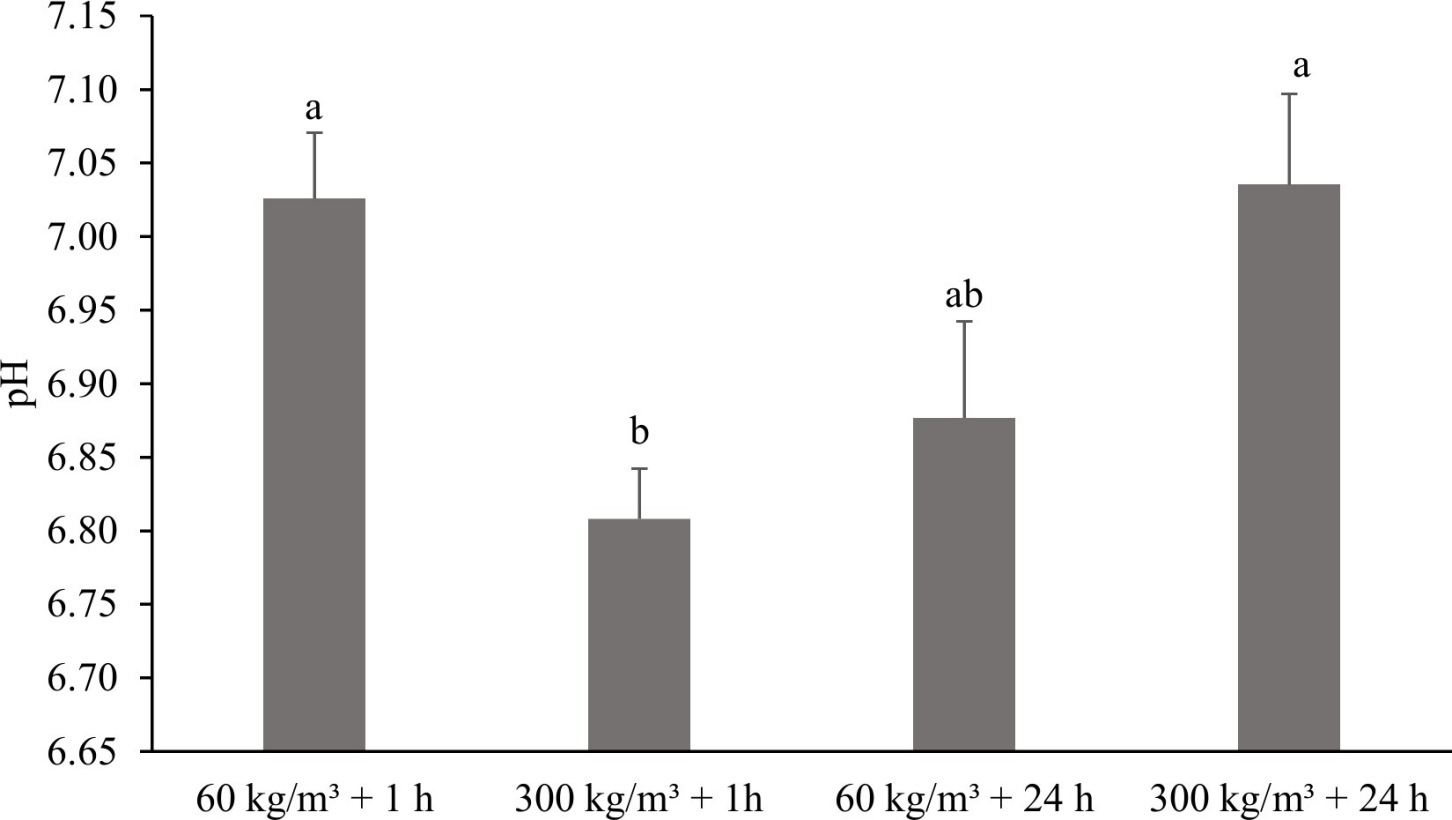
pH values of Nile tilapia fillets at different densities (60 and 300 kg m^-3^) and depuration times (1 and 24 hours). Lower case letters indicate significant difference (P = 0.0027) by Tukey’s test. Vertical bars indicate standard error of the mean.

For pH, there was an effect of the interaction between density and depuration time (P = 0.0027) where the treatment with 300 kg m^-3^ and depuration for 1 hour resulted in fillets with lower pH than the others (Fig 2). When the factors were analyzed individually, no effect of density (P = 0.5846) or depuration time (P = 0.4738) was observed.

The color parameters (L*, a* and b*), shear force and water-holding capacity are presented in Table 3. For lightness (L*), there was no effect (P = 0.3843) of the interaction between density and depuration time. However, when the factors were analyzed separately, higher density (300 kg m^-3^) and higher depuration time (24 hours) resulted in fillets with a higher mean L* value. Likewise, for chroma a*, there was also no effect (P = 0.1879) of the interaction between density and depuration time. However, when evaluating these factors separately, the density of 300 kg m^-3^ produced fillets with higher (P = 0.0003) mean redness intensity (a*). For yellowness (b*), no differences (P>0.05) were observed for the interaction density *versus* time nor for the individually analyzed factors.

**Table 3:** Color parameters (L*, a * and b *), shear force and water-holding capacity (WHC%) of Nile tilapia fillets at different densities (60 and 300 kg m^-3^) and depuration times (1 and 24 hours).

Density (kg m^-3^)	Time (h)	L *	a*	b*	Shear force (gf)	WHC%
60	1	46.47±0.69	-2.09±0.11	0.67±0.39	16372 ± 272 a	59.47±0.56
	24	47.82±0.82	-1.78±0.07	1.04±0.20	15518 ± 1321 a	59.19±0.53
300	1	47.77±0.43	-2.52±0.20	1.23±0.30	11390± 475 b	59.59±0.53
	24	50.36±0.78	-2.56±0.12	0.36±0.42	14580 ± 576 ab	59.03±0.45
Density (kg m^-3^)						
60		47.14±0.55 b	-1.93±0.08 a	0.85±0.21	15945 ± 652 a	59.32±0.38
300		49.06±0.60 a	-2.54±0.11 b	0.79±0.28	13162± 667 b	59.33±0.35
Time (h)						
1		47.12±0.44 b	-2.31±0.13	0.95±0.25	14158 ± 908	59.54±0.38
24		49.09±0.68 a	-2.17±0.15	0.70±0.25	15049 ± 697	59.11±0.34
P values						
Density versus time effect		0,3843	0,1879	0.0820	0.0239	0.7879
Density effect		0.0139	0.0003	0.8616	0.0138	0.9748
Time effect		0.0120	0.3192	0,4708	0.5150	0.4209

L *: brightness (L* = 0 black and L* = 100 white), chroma a* (red-green component) and chroma b* (yellow-blue component). Means in the same column followed by different letters differ from each other by Tukey’s test. Data are expressed as the mean ± standard error.

For tenderness (expressed by shear force), there was a significant interaction (P = 0.0239) between density and depuration time where 300 kg m^-3^ for 1 hour resulted in more tender fillets compared to other treatments. When the factors were analyzed separately, the density of 60 kg m^-3^ led to a significantly (P = 0.0138) shear force greater than compared to the density of 300 kg m^-3^.

The water-holding capacity of the fillets was not affected by the interaction between density and depuration time (P = 0.7445) nor the individual factors (P = 0.9043 for density; and P = 0.1648 for depuration time).

### Sensory analysis

In the sensory profile of the fillets of tilapia subjected to different densities (60 and 300 kg m^-3^) and depuration times (1 and 24 hours), no effect of the interaction (P>0.05) between density and time was observed for any of the sensory parameters evaluated (color, texture, juiciness and general acceptability) (Table 4). However, the fillets of tilapia subjected to a density of 300 kg m^-3^ presented less general acceptability compared to the fillets of tilapia subjected to a density of 60 kg m^-3^ (P = 0.035).

**Table 4:** Sensory profile and correlation matrix between the attributes of Nile tilapia fillets at different densities (60 and 300 kg m^-3^) and depuration times (1 and 24 hours).

Density (kg m^-3^)	Time (h)	Color	Texture	Juiciness	Overall Acceptability
60	1	7.13±0.14	7.54±0.12	7.35±0.14	7.29±0.13
	24	7.24±0.14	7.58±0.11	7.55±0.13	7.57±0.11
300	1	7.02±0.14	7.37±0.13	7.23±0.15	7.25±0.13
	24	6.97±0.15	7.40±0.16	7.27±0.16	7.27±0.14
Density (kg m^-3^)					
60		7.18±0.10	7.56±0.08	7.45±0.09	7.43±0.09 a
300		6.99±0.10	7.38±0.10	7.25±0.11	7.26±0.09 b
Time (h)					
1		7.07±0.10	7.45±0.09	7.29±0.10	7.27±0.09
24		7.10±0.10	7.49±0.10	7.41±0.10	7.42±0.09
Effects					
Density vs. time		0,433	0.965	0.595	0,191
Density		0.070	0.094	0.064	0.035
Time		0.797	0.761	0.355	0,137
Correlation matrix					
		Color	Texture	Juiciness	Overall Acceptability
Color		**1**	0.912	0.934	0.863
Texture			**1**	0.879	0.714
Juiciness				**1**	0.960
Overall Acceptability					**1**

Hedonic scale between 1 (disliked extremely) and 9 (liked extremely); Data are expressed as the mean ± standard error of the mean.

Considering the Pearson correlation coefficients among the four attributes of the tilapia fillets analyzed (Table 4), a positive correlation was observed among all attributes with acceptability being more related to fillet juiciness (0.960).

**Fig 3:**
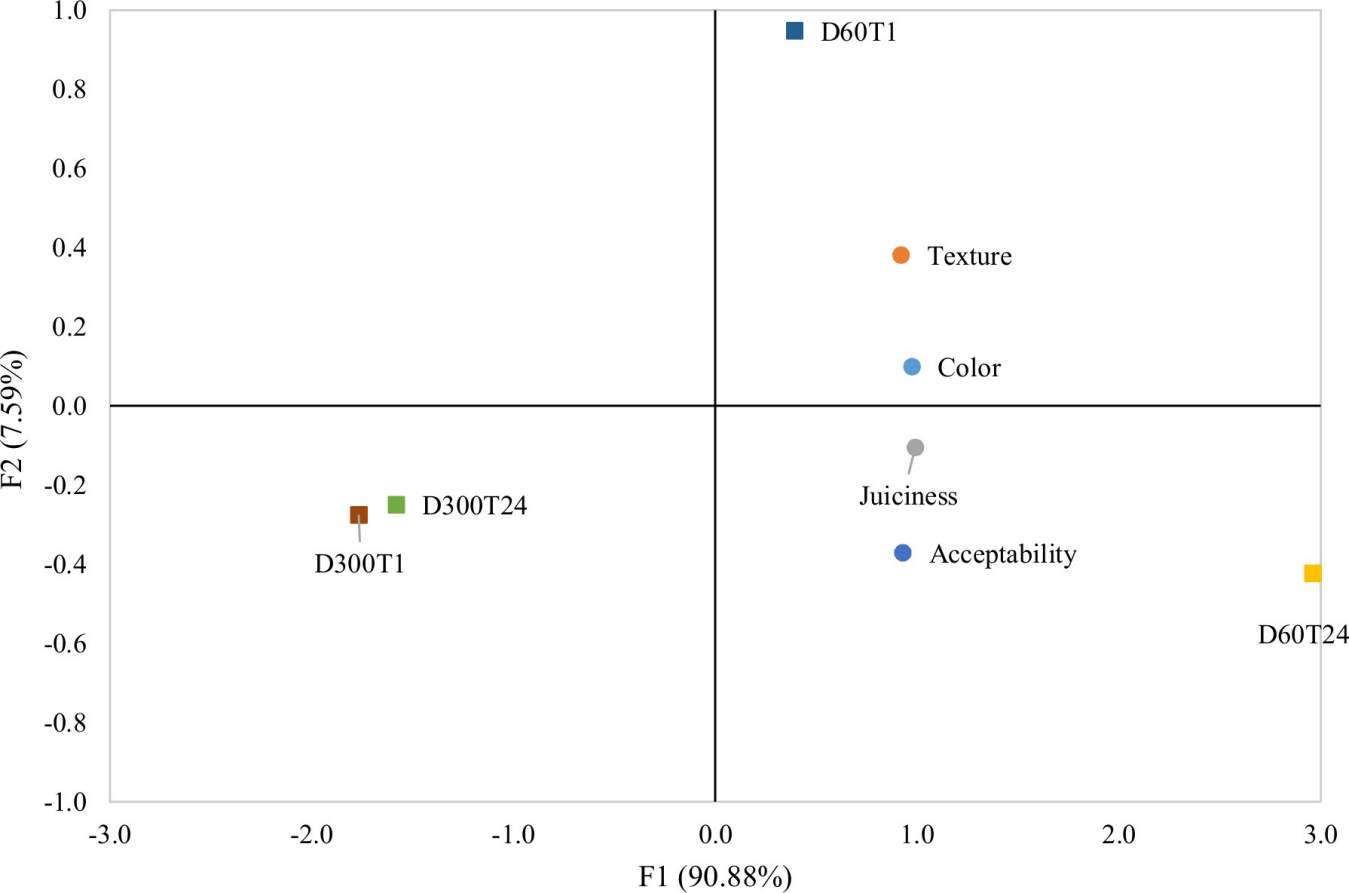
Principal components analysis. D60T1, density of 60 kg m^-3^ for 1 hour; D60T24, density of 60 kg m^-3^ for 24 hours; D300T1, density of 300 kg m^-3^ for 1 hour; and D300T24, density of 300 kg m^-3^ for 24 hours.

In the PCA (Fig 3), the two principal component axes explained 98.47% of the total variance. Regarding the treatments, the attributes of texture, color, juiciness and acceptability were positioned on the right side of F1 located close to treatments D60T24 and D60T1. The other treatments (D300T24 and D300T1) were placed on the other side (F1 left) inversely related to the analyzed attributes. Texture and color were present in the same quadrant as D60T1, demonstrating an association between them, and the same result occurred for juiciness and acceptability with the D60T24 treatment. Thus, PCA demonstrated that the fish at lower density (60 kg m^-3^) were associated more with the sensory attributes evaluated.

## Discussion

In fish, acute stress exposure causes rapid elevation of cortisol levels, which are quickly restored to resting levels during recovery from stress [21]. In the present study, the increase in the density of tilapia for a short period of time increased serum cortisol levels regardless of the density being high or low. However, the maintenance of fish at low density allowed recovery from stress over time, which did not occur at high density. In general, plasma cortisol levels increase rapidly after exposure to acute stress, and normal conditions are restored within a few hours [22].

The maintenance of tilapia at high density for a long period of time apparently led to a chronic stress condition because the cortisol level in fish at a density of 300 kg m^-3^ for 24 hours was higher than the others. High fish densities can affect fish performance and well-being through crowding stress and/or changes in water quality [23]. Chronic stress usually involves changing the energy metabolism to deal with the stressor agent, which significantly affects the immune system of the animal [24].

During stress, elevated levels of plasma cortisol mobilize energy stores primarily through genomic actions [25]. In the present study, the expression of the CAT and GPx enzymes was similar in both liver and muscle. The fish subjected to the lower stocking density and the shortest depuration time had the highest CAT and GPx expression levels. The activity of these enzymes is an important indicator of the activation of the cellular antioxidant defense system and protection against oxidative stress [26]. Oxidative stress is the imbalance between the production and degradation of oxygen reactive species (ROS), such as superoxide anion, hydrogen peroxide and lipid peroxides [27]. The enzymatic inactivation of ROS in muscle tissue is performed mainly by the superoxide dismutase (SOD), CAT and GPx enzymes [8]. The decrease in enzyme activity may be related to alteration or reduction of gene expression and transcription [27]. In the present study, the most stressed animals (at a density of 300 kg m^-3^) presented lower expression of the CAT and GPx enzymes. Oxidative stress may have led to a decrease in the activity of antioxidant enzymes as suggested by Kalmar and Greensmith [14]. In agreement with our results, a reduction of antioxidant enzyme activities has been observed in stressed pigs [28] and in broilers [27] [11] compared to non-stressed animals.

The higher stress caused by high density resulted in lower expression of the CAT and GPx enzymes, which may have generated more tender fillets. The oxidative stress was related to the process of meat softening [12] due to greater proteolysis and protein oxidation [11]. Oxidation leads to protein fragmentation or aggregation and decreased protein solubility, which affects meat quality [8]. Protein oxidation may also play a role in controlling the proteolytic activity of enzymes and may be linked to meat tenderness [9]. PSE chicken meat has lower CAT, GPx and SOD activity than normal meats [11]. Picard et al. [29] reported that enzymes involved in oxidative stress, such as SOD or peroxiredoxin 6 (PRDX6), are negatively related to tenderness.

Changes in meat tenderness may also be related to the activation of heat shock proteins (HSPs) in the muscle. In the present study, animals subjected to high density stress (300 kg m^-3^) showed higher HSP70 expression and more tender fillets. In response to cell stress, such as hyperthermia, oxidative damage, physical injury or chemical stressors, the expression of HSPs increases dramatically [14]. HSPs delay the rate of muscle aging and decrease the degradation of myofibrillar proteins [30]. Studies on beef have identified HSPs as biomarkers of meat tenderness, and HSP activity differs between tender and tough meat [31] [32] [33] [34]. The lower activity of HSP70 is associated with higher beef tenderness [33] [12] [34]. In contrast, in the present study, fish with higher HSP70 expression (high density = greater stress) produced fillets with a less firm texture. Thus, the higher HSP70 expression was apparently not enough to slow protein oxidation, demonstrating that this chaperone is not as efficient in fish as it is in beef. According to Ouali et al. [35], HSPs contribute to meat tenderness through the following mechanisms: i) forming a complex with active caspases, thus hindering their function; ii) protecting target proteins (substrates) from caspases that prevent or delay their degradation; iii) trying to reestablish the initial and active structure of proteins that underwent structural damage after stress or the beginning of apoptosis; and iv) triggering apoptosis when the stress is no longer sustainable.

Another mechanism associated with decreased firmness in fillets may be pH decline. Fish subjected to high density (300 kg m^-3^) for 1 hour produced fillets with lower pH and shear force. Vigorous swimming under stress conditions leads to intense white muscle use, increasing anaerobic glycolysis and lactic acid production, which leads to a reduction in muscle pH [36] [37]. Slaughtering fish stocked at high density (300 kg m^-3^) after 1 hour may have resulted in a faster use of glycogen with an increased anaerobic respiration rate, resulting in higher production of lactic acid and lower pH of the meat. The pH decline post-mortem may negatively affect the texture of fillets as it alters protein solubility and increases the proteolysis and denaturation rate [38].

High density (300 kg m^-3^) for 24 hours resulted in higher pH, which may be related to the depletion of glycogen reserves. The intense activity for a long period of time before slaughter causes the fish suffer great wear and can deplete glycogen completely. The high consumption of glycogen by stress and the simultaneous removal of lactic acid by the circulatory system in the living animal would leave it without reserve of glycogen, so that, after death, rigor mortis would proceed without production of lactic acid, with the pH remaining high, resulting in the absence of the pre-rigor phase and a full rigor without decreasing pH, called alkaline rigor mortis [39]. In salmon (*Salmo salar*), 24 hour stress leads to the production of meat with higher pH and firmer texture, which is attributed to the severe depletion of glycogen stores [40].

High density stress also led to the production of fillets with higher lightness and lower redness. More stressed fish fillets can develop with higher brightness, and changes in color. This may be related to a change in pH caused by stress, which induces a faster denaturation of the protein and therefore a change in the pattern of light reflection in the muscle, an effect established quite early in the rigor process [41]. These hypotheses were corroborated in the present study, which evidenced the relationship between stress arising from higher density and the development of changes in fillet color. Other studies with fish have also reported an increase in lightness after exposure to acute pre-slaughter stress [37] [42] [41].

The expression of HSP70 may also have affected the tilapia fillet color. In pork muscle subjected to transport stress, a decrease in the activity of HSPs impairs the integrity of muscle cells and the repair of denatured proteins, leading to reduced flesh color and WHC [15].

Changes in the quality of fish fillets subjected to high density stress resulted in losses in the sensory acceptance of fillets because tilapia at the density of 300 kg m^-3^ presented fillets with less general acceptability. In the correlation analysis, the acceptability was more related to the juiciness of the fillets. A previous study has shown that less stressed tilapia produces meat with higher WHC, lower water loss by pressure and higher juiciness [43]. In the present study, the lower acceptance may be related to the changes observed in the instrumental quality (greater tenderness, greater lightness and lower redness). In fish, the best quality is firm and cohesive flesh with good water holding capacity [29]. Therefore, excessive fillet tenderness is highly undesirable as it can have a major impact on consumer acceptance [44].

It should be noted that fish fillets subjected to lower density (60 kg m^-3^) were more associated with the sensory attributes evaluated, while the fillets from more stressed fish (300 kg m^-3^) were inversely related to the attributes analyzed as demonstrated by the PCA. Likewise, a previous study with cod has also shown that less stressed fish obtain higher scores on the attributes of general acceptance, texture and juiciness [45].

Therefore, high density generated oxidative stress, decreased the expression of antioxidant enzymes (CAT and GPx) and increased the expression of HSP70 in tilapia. These changes negatively affected the quality of the fillets, which presented less firm texture, greater lightness, less redness and less sensory acceptability. To our knowledge, these are the first data on the link between redox imbalance and deleterious changes in fish meat quality. These results indicated stress should be controlled in the pre-slaughter period of fish, aiming to improve the quality of the meat and the lives of the animals. Although the maintenance of fish at high density is feasible, it can cause sensory damage to the quality of fish fillets.

## Conclusions

High density (300 kg m^-3^) during tilapia pre-slaughter causes lower expression of the CAT and GPx enzymes but higher expression of HSP70, resulting in the production of fillets with higher tenderness, higher lightness, lower redness and decreased sensory acceptability. Low density and longer depuration time are recommended for decreased stress and improved quality of tilapia fillets.
